# How Can We Increase the Nutrition-Related Knowledge in Children Aged 7–12 Years: Results of Focus Groups Interviews with Parents—Junior-Edu-Żywienie (JEŻ) Project

**DOI:** 10.3390/nu16010129

**Published:** 2023-12-30

**Authors:** Ewa Czarniecka-Skubina, Jadwiga Hamulka, Krystyna Gutkowska

**Affiliations:** 1 Department of Food Gastronomy and Food Hygiene, Institute of Human Nutrition Sciences, Warsaw University of Life Sciences (SGGW-WULS), 166 Nowoursynowska Street, 02-787 Warsaw, Poland; ewa_czarniecka-skubina@sggw.edu.pl; 2 Department of Human Nutrition, Institute of Human Nutrition Sciences, Warsaw University of Life Sciences (SGGW-WULS), 166 Nowoursynowska Street, 02-787 Warsaw, Poland; 3 Department of Food Market and Consumer Research, Institute of Human Nutrition Sciences, Warsaw University of Life Sciences (SGGW-WULS), 166 Nowoursynowska Street, 02-787 Warsaw, Poland; krystyna_gutkowska@sggw.edu.pl

**Keywords:** parents of pupils, parental attitudes, nutritional knowledge, dietary habits, nutritional education, Focus Group Interview (FGI)

## Abstract

Nutrition education is a long-term process that should cover various population groups. A special focus should be placed on children, adolescents and their parents. The aim of this research was to find out the opinions of parents of primary school pupils aged 7–12 on their expectations towards school education in the areas of food and nutrition, addressed to both pupils and their parents. The research was conducted among 101 parents of primary school pupils with the use of the Focus Group Interview (FGI) method. It demonstrated that what is most needed are hands-on activities relating to basic theoretical issues. While parents see the need for nutrition education for their children, educating pupils in this area is of interest to only some of the respondents for whom nutrition aspects are quite important. All parents would like formal nutrition education at school, but at the same time, they do not want classes to take up too much of their children’s time, due to the already excessive number of school subjects. It seems appropriate to include everyone in regard to nutrition education, regardless of their declared interest in this issue. The need for consistent presentation of educational content addressed to teachers and parents is very important, so that they can, in a uniform way, shape the attitudes towards food and nutrition of children and adolescents.

## 1. Introduction

Nutrition education is a long-term, lifelong process based on the systematic transfer of knowledge about food and nutrition, based on current scientific research results, the effect of which should be a change in eating habits [1]. In order to achieve this aim, this education should cover all population groups, especially children and young people. Parents are also an important group, due to the fact that they are role models for their children and have responsibility for their early socialization [2]. Therefore, designing and implementing programmes supporting the development of eating skills from early childhood contribute to the formation of more health-promoting and sustainable eating habits in entire societies [1].

Previous studies have demonstrated the impact of parents’ eating habits on their children’s eating behaviours, regardless of their sociodemographic characteristics such as gender, age, socioeconomic status or country of residence [3]. Children, observing their parents, imitate their eating behaviour, especially when meals are eaten together [4,5,6,7,8,9]. As a result, children can recognise and perceive their parents’ behaviour as appropriate and even exemplary in terms of consumption of given food products [10]. Therefore, parents’ dietary habits have a crucial influence on their children’s dietary habits. It is parents who shape the domestic nutrition environment, decide on the availability of consumed products, influence children’s views through their own eating preferences and behaviours, i.e., through the frequency of eating meals, the inclusion of breakfast and eating vegetables and fruit, but also through restrictions on the consumption of sweets, fast food or salty snacks [3,11,12,13,14]. Parents’ eating behaviours are part of the entire process of shaping and promoting both correct and incorrect eating patterns among children and adolescents [4]. Moreover, it should be noted that authoritative orders are the wrong format for informal nutrition education that takes place in the family. Therefore, obliging a child to eat ”healthy” food by giving them only advice, without a proper example from parents, will not yield long-term results [15].

Family meals constitute an important element of control and interaction between parents and their children [3]. The environment in which the meals are eaten and the fact that they are prepared at home are also important, especially in those countries where eating out is popular [16]. The study The Identification and prevention of Dietary- and lifestyle-induced health Effects in Children and infants (IDEFICS), in which families (*n* = 1435) from eight European countries took part, demonstrated that the home food environment plays a greater role in shaping children’s healthy food consumption habits than unhealthy food available outside the house; this is especially true for younger children [17].

Moreover, the influence of parenting practices on children’s eating behaviour depended on the children’s age. The greatest impact was noted in the case of children of preschool age and those in the first years of primary school [10]. In the case of teenagers, a number of additional factors should also be taken into account, such as the influence of the peer environment and adolescents’ concern for their own body image and health, as well as barriers and motivations for making nutrition choices, including food purchases [18,19]. In this case, despite less control on the part of parents, the family environment can and should provide even more support for children’s final decisions and nutrition behaviours, and educational strategies should take into account many factors at the same time. The literature emphasises the importance of maintaining a balance between promoting a healthy lifestyle while maintaining sensitivity to teenagers’ practical and social issues, as well as those related to body image [18,20]. This is particularly important due to the fact that currently, the mass media promote the model of a slim figure in women and an athletic figure in men, advertising food products and diets with inadequate nutritional value, which may also lead to undesirable eating and health behaviours and non-acceptance of one’s own body weight, especially among teenagers [21,22]. Moreover, it is important to promote home-cooked meals, as many authors emphasise that eating food that is not prepared at home is associated with poorer diet quality, greater consumption of meals high in fat and sugar and with a lower content of vitamins and minerals, and generally consumption of high-calorie food [23,24,25].

It has been stated that early changes to more healthy eating habits in childhood can promote health and reduce the risk of developing diet-related diseases later in life [3].

Taking into account the wealth of scientific evidence [2,10,26,27] regarding the influence of parents on shaping the eating habits of their children, an attempt was made to identify the needs of parents in the field of nutrition education of children and for themselves, so that they can improve their own diet and correct their own improper behaviour as well as that of their children, in order to build a consistent image of proper nutrition. By conducting qualitative research using the FGI method, it was possible discover parents’ spontaneous statements about food and nutrition and their ideas about nutrition education.

Considering that, to the best of our knowledge, there has been little research work using FGI in this field, this research was undertaken. The aim of this study was to find out the opinions of parents of primary school pupils aged 7–12 on their expectations towards school food and nutrition education addressed to pupils and the nutritional education of parents.

## 2. Materials and Methods

### 2.1. Study Design and Participants

The Focus Group Interview (FGI) technique was applied to conduct qualitative research among Polish parents of primary school students aged 7–12 years. The individual groups of parents were divided into groups of parents of children aged 7–9 years (*n* = 47) and parents of children aged 10–12 years (*n* = 54), which resulted from differences in the development and teaching of these groups of students. Data were collected in 10 locations all over Poland, including three environments: big cities (Warsaw, Białystok, Lublin, Kielce), small towns (Ostrowiec Świętokrzyski, Nowy Sącz, Brańszczyk) and rural (Rosko, Czachówek, Poręba). Each focus group consisted of 6–7 parents, and 16 group interviews were organized. A total of 101 parents from schools that signed up for the JEŻ project (*n* = 2218) participated in the study. More details on the study design and methods were described previously [28,29]. The study was carried out as part of the Junior-Edu-Żywienie (JEŻ) project.

### 2.2. FGI Moderation

The research method was the Focus Group Interview among parents, who freely expressed their opinions on food and nutrition issues following a specific moderation scenario prepared by the authors of this article. The moderator leading the discussion encouraged participants to exchange ideas and engage in the discussions [10,30,31,32,33]. 

The structure and substantive content of FGI are included in Supplementary File S1. The FGI moderation scenario was tested in a pilot study. The study was conducted by three moderators, one of whom recorded the interviews (with the participants’ permission) and took notes. After introducing himself, the moderator asked the participants to introduce themselves, and then he presented the idea of the research and explained the rules of group behavior during the discussion (confidentiality, respecting each other’s opinions, not interrupting each other, relevance/importance of each opinion presented in the study).

The research focused on parents’ opinions regarding acquiring nutrition knowledge by pupils and the current state of children’s nutrition education and needs in this area, as well as parents’ ideas on how this education should be implemented. The qualitative research was conducted by the professional company, Umbrella Agency Marketing Group. Each FGI lasted approximately 90 min.

Inclusion criteria for the focus group were being a parent or legal guardian of student aged 7–12, willingness to participate and written consent to participate in the study. More details about this study are presented in our other article [29]. 

### 2.3. Procedures and Data Analysis

Interview transcripts and additional notes recorded during moderation were coded and preliminarily analyzed by two independent researchers, and any connections were discussed to reach consensus. In the results analysis, only the town or city name where the study was carried out was assigned to the parents’ statements.

The material obtained in FGI was analyzed according to the principles of grounded theory, with an emphasis on discovering recurring and visible themes that resonated throughout the discussions [34]. A seven-step approach for data analysis was applied, including: (1) familiarization with the data, (2) coding according to themes, (3) sub-theme identification within the main framework, (4) sub-theme revision, (5) definition and naming of each sub-theme, (6) analysis and interpretation of patterns throughout the data area, and (7) combination of sub-themes into dominant contextual domains.

### 2.4. Ethical Approval

This study was approved by the Ethics Committee of the Institute of Human Nutrition Sciences of the Warsaw University of Life Sciences (No. 18/2022). All of the participants provided informed consent to participate in the study. The Interview format was explained to the participants before the start. Participants could resign from participation in the FGI at any stage, without giving any reason; however, in this case, such a situation did not occur.

## 3. Results

### 3.1. Sources of Nutrition Information among Pupils from the Parents’ Perspective

Parents of pupils aged 7–9

The vast majority of parents (*n* = 41) of pupils aged 7–9 admitted that their children gain knowledge on topics that interest them mainly from home, school and peers. The Internet is also a very important source, although it is more a source of entertainment than a source of knowledge or specific information. Most parents (*n* = 33) also declared that they limit the time their children spend in front of computer screens, smartphones or other such devices, especially since most children use their parents’ equipment and with their consent, mainly during weekends. It should be mentioned that parents of younger children use so-called parental controls and do not have to track the content searched by their children on the Internet, because their children do not have access to certain websites. Therefore, they were able to indicate general names of websites or sources used by their children, but were unable to indicate specific names of channels, websites, profiles, etc.

According to the parents, their children most often use YouTube, and it is the main source for watching funny videos (uploaded by creators), cartoons (watched more often by girls), sports activities (mainly of football players, most often watched by boys) and for observing players playing online games, e.g., Minecraft (also more often watched by boys). For pupils in this age, influencers are of little importance.

Pupils of this age group use social media, e.g., Facebook and Instagram, to a very limited extent; those that do are mostly children from larger cities, but these are isolated cases. TikTok is also a source of interesting, funny, short clips, mainly for children from larger and medium-sized cities, whose parents or legal guardians have this app installed on their mobile phones.

Teachers are also an important source of knowledge for younger pupils. In their relations and conversations with their parents, children often refer to their teachers’ opinions. Therefore, according to the study participants, if a campaign on healthy eating is organised, teachers should also take part in it:

*“Teachers should also sometimes take out a sandwich and show what they’re eating. If we check lunch boxes, it should apply to everyone!”*

(parents of pupils aged 7–12, Warsaw)

On the other hand, according to some parents (*n* = 30) participating in the study, grandparents are often anti-role models, and this opinion was expressed by parents with higher nutritional awareness. In their opinion, children should not follow their grandparents’ example because their approach to nutrition reflects many myths and is often wrong, e.g., they eat a lot of fatty foods, allow sweets at any time of day, and the amount of vegetables in their diet is often negligible. They also often reward children with their favourite sweets or snacks.

Sample statements by pupils’ parents:

*“It’s not possible to explain it to grandparents. They behave according to their traditional rules, which are difficult and sometimes impossible to change. During the main meal of the day, they give a child sweets between one course and the next.”*

(parents of pupils aged 7–9, Czachówek)

Parents of pupils aged 10–12

It was sometimes difficult for parents to admit that they were recommending that their children follow a healthy diet without setting an example or following it themselves. It was similar in the case of sports, movement and physical exercise. During the discussions, a lack of coherence was often noticed between the rules expected to be followed by children and the actual behaviour of parents in this respect. As parents observed, pupils aged 10–12, being keen observers, quickly notice this type of discrepancy and, as a result, they easily rebel and express their opinion, which they are even more willing to do when their parents give them reasons to do so.

The vast majority of parents (*n* = 42) of pupils aged 10–12 believe that children gain knowledge about nutrition mainly from the Internet. Most parents allow teenagers more freedom in using tablets, smart phones and computers. They emphasise, however, that nowadays, children also do their homework using the Internet, and this medium is also used for communication with their peers, so it is impossible to completely prohibit children from using this tool.

It was difficult for parents participating in the study to indicate specific names of channels, profiles, websites or Internet content creators that their children like and follow. The respondents indicated that their children certainly use YouTube, which is the main source of funny videos, curiosities and content posted by creators, including in the field of nutrition. YouTube is also starting to serve as a source of knowledge. If they are interested in a certain topic (e.g., sports, nutrition, food, drawing, travelling, animals), they explore it by watching videos and following so-called themed channels. They receive knowledge presented in a short format, and they decide for themselves what is of interest to them and what isn’t, and they “click” in search of content interesting for them.

For children of this age, influencers also start to be an important source of inspiration, knowledge and information, although parents were unable to point to specific examples of such people. Just as in younger age groups, in older groups, TikTok is used mainly as a source of interesting, funny, short clips. Parents admit that the impact of influencers among older pupils is enormous. They want to wear clothes, eat products or buy gadgets promoted by influencers online. According to most parents, the impact of Internet content on children is irrational: they cannot distinguish between what is valuable and what is not, instead assuming that all information presented there is reliable.

Sample statements by pupils’ parents:

*“My son often watches videos posted by YouTubers. They travel around and test food. One day they eat fast food, other times they eat salads, and evaluate which one is cool, which one is good, which one is bearable, which one is bad. And if a certain YouTuber says that this one is good, then according to the kids it definitely is good! It doesn’t matter if their mum makes the same one at home. The other one is definitely better.”*

(parents of pupils aged 10–12, Poręba)

*“I have the impression that sometimes they don’t take into account whether it is a bad or good example, whether something is bad or good. Sometimes they do not differentiate such things.”*

(parents of pupils aged 10–12, Brańszczyk)

Older pupils also begin to actively use social media, e.g., Facebook and Instagram. In the opinion of parents participating in the study, this is one of the ways for a child to have contact with peers and not be ”excluded from the group”. Parents from larger cities expressed more relaxed opinions about the use of social media by pupils aged 10–12. This is probably due to the fact that children in smaller towns or rural areas live close to each other and find it easier to meet than to use social media, and they do not copy their parents using them either.

According to parents (*n* = 38), it is important to note that children of this age, just like younger children, are willing to share with their parents the knowledge they have acquired from the Internet, and what interesting things they have watched, seen or read. Parents—more often men—participating in the study admitted that they like to watch various videos with their children, both funny and knowledge-providing. The Internet is therefore a space for spending time together, having fun and learning.

Almost half of parents stated that peers and teachers are also an important source of knowledge on nutrition for older pupils. However, it should be borne in mind that the influence of the former increases with pupils’ age, while the influence of the latter decreases as pupils age. Parents are aware that at this age, friends play a huge role in many areas.

Sample statements by pupils’ parents:

*“We may think that a child doesn’t do something, or doesn’t eat or drink what we forbid them to do, but they behave differently among their peers and we may not even know about it.”*

(parents of pupils aged 10–12, Lublin)

*“Your parent tells you: eat this, eat that. So he resists, but it looks different in his peer group. Someone has eaten something, he’s fine, so maybe nothing will happen to me. My friend has eaten it, so I’ll eat it too. As the saying goes: it tastes better at someone else’s house.”*

(parents of pupils aged 10–12, Białystok)

### 3.2. Key Aspects of Nutrition Education of Children

The most important aspects of nutrition education which, from the point of view of parents of pupils aged 7–9 and 10–12, should be included in the school curriculum are presented in Table 1. The importance of these issues was determined by asking the study participants how important each aspect was to them and asking them to respond using a scale of 1–5. Their answers were counted, and the average levels of importance are shown in the Table 1.

It can be noticed that themed areas within nutrition education and their importance for both younger and older pupils are similar. Parents believe that children should learn a wide range of topics from an early age, but the level of detail should change with age. In older year groups, additional topics related to puberty, physicality and aspects related to the impact of nutrition on a child’s appearance are expected. It is equally important to discuss the dangers associated with energy drinks. According to a few parents, education of pupils aged 7–9 should also include this aspect, because many of them have older siblings.

Some of the interviewed parents of pupils aged 7–9 and 10–12, especially those from larger cities and those more interested in nutrition, expressed the opinion that the current curriculum lacks issues related to ethical aspects associated with food and nutrition, referring to:

1. zero waste: currently, this is a very important topic, and children should be made aware from the first school years why food should not be wasted and how to prevent waste;
2. vegetarian/vegan diet: their role and importance for environmental protection, possible meat substitutes and protein sources;
3. food production method vs. quality: how to recognise quality, how to make rational purchases, the role of certificates and markings on products (e.g., free-range eggs, etc.), different ways of producing food products (small-scale production vs. mass production or breeding).

These topics are particularly important according to those parents who are aware of proper nutrition and those who try to practise a healthy lifestyle, including healthy eating.

### 3.3. The Scope and Implementation of Nutrition Education at School in the Opinion of Parents 

All parents of pupils (aged 7–9 and 10–12) participating in the study confirmed that they notice the impact of early school and even preschool education on their children’s knowledge of nutrition. They even stated that thanks to nutrition education, children have solid basics of knowledge based on the understanding of a food pyramid, the health-promoting importance of the consumption of vegetables and fruit, and limiting the consumption of products that are harmful to health, mainly sweets, fats, salty snacks and fast food. 

Sample statements by pupils’ parents:

*“There was a time when they had a food pyramid at school and it had some impact on them, because, for example, during meals, my children would point out that they needed to add something else to the meal because it was at the top of the pyramid.”*

(parents of pupils aged 10–12, Poręba)

Opinions of parents of pupils aged 7–9

However, in the perception of this study’s participants, the scope of nutrition education should be much wider than that implemented so far and should change and expand alongside children’s age and knowledge level. According to the pupils’ parents, the most important thing is consistency and continuity of this process, thanks to which there is a chance to systematically consolidate children’s knowledge and nutritional awareness. According to the respondents, talks during general educational classes and occasional meetings with specialists or experts in the field of nutrition are valuable, but children treat them as an interruption during their classes and a temporary distraction. Meanwhile, from the early school years, pupils should understand and feel the importance and significance of proper nutrition through learning and fun during hands-on classes and workshops.

A significant group of parents (*n* = 40)—regardless of the type of location—declared that nutrition could even be a separate subject taught once a week. However, they are concerned that children would be burdened with more learning and would spend too much time at school. Another option expected by the vast majority of parents is a regular schedule, e.g., once a month or at least once every three months, of additional teaching hours or activities during the children’s stay in a school club. Most parents of younger pupils expressed the opinion that these classes should take place throughout the entire teaching period and be adapted to pupils’ level of understanding.

Sample statements by pupils’ parents:

*“If these classes take place once a week at school and a child comes home and sees a million TV ads encouraging consumption of sugar and processed food, it is difficult to prove the attractiveness of celery or salad over crisps. It’s a bit of a no-win situation from the start, because the attractiveness of the form wins and that’s where the problem lies.”*

(parents of pupils aged 7–9, Nowy Sącz)

*“It depends on how often and when. If these were to be just one-off workshops, I’m not convinced that it would have the desired effect. They would have to be held periodically.”*

(parents of pupils aged 7–9, Brańszczyk)

Parents of pupils aged 7–9 (*n* = 38) who participated in the study agreed that nutrition education classes should be as practical as possible. In their opinion, children should “learn by touching food products, having fun playing with them and making their hands dirty” as a result, and at the end, they should try what they have prepared to eat themselves. During such classes, there should also be space for children to acquire theoretical knowledge and ask questions, rather than just listen. Making such classes interactive and practical would help children remember the material that is introduced.

The study participants also mentioned that their children like to take part in competitions and games because winning a prize motivates them to make an effort and get involved. Experiences, demonstrations and experiments seem to be important forms of activity, e.g., how many sugar cubes are in a glass of various types of drinks or showing the energy value of food products. In the perception of parents of younger pupils, such a combination and variety of theoretical and practical classes would yield the best results in the field of nutrition education. Hands-on classes also teach children how to prepare meals by themselves and develop manual skills (e.g., cutting, peeling).

It was observed that it was difficult for the study participants to determine who should be responsible for conducting nutrition education at school. Should it be a headteacher, tutor, nurse, or an external body/institution? From their perspective, this is a secondary issue; what is more important is that whoever organises such classes would need specialist knowledge, enthusiasm and ideas on how to plan and organise them. Parents of pupils aged 7–9 also wanted nutrition education at school to be imposed by the Ministry of Education and Science or school governance councils. Thanks to this, the curriculum would be unified and consistent in all schools and developed in a professional manner, but also taken seriously by pupils and parents themselves.

Sample statements by pupils’ parents:

*“It should be a nationwide programme. It should be implemented in the same way in every school. Most importantly, these classes would have to be conducted by people who have the knowledge, skills and, generally speaking, authorisation to teach such classes in schools.”*

(parents of pupils aged 7–12, Warsaw)

*“In my opinion it should be someone from outside the school. Someone who knows all about it. Someone who has ideas. For example, I’m a teacher and what do I know about nutrition? Why should I play the role of an educator in every possible field?”*

(parents of pupils aged 7–9, Ostrowiec Świętokrzyski)

*“It would be best if it was an educational programme. Let someone who has an idea or scripts or whatever is needed to handle it. So that it all makes sense. Because if it’s detached from reality, it will look more or less the same, as always.”*

(parents of pupils aged 7–9, Białystok)

*“By banning something, we won’t get positive results. There must be examples, preferably practical ones.”*

(parents of pupils aged 7–9, Nowy Sącz)

Individual parents (*n* = 12) from smaller towns expressed the need to seek advice from a dietitian at a free school clinic. This mainly applies to children struggling with nutritional problems, e.g., obesity, low body weight, food intolerances, or eating disorders.

Opinions of parents of pupils aged 10–12

According to most parents of older pupils (*n* = 48), nutrition education is not as thorough as it is in case of younger pupils. The biology and technology curriculum includes several lessons devoted to developing the principles of healthy eating habits.

However, according to the study participants, the scope of nutrition education should be much broader and spread over a longer period of time, and should change and expand with children’s age and level of knowledge. In the opinion of most of the parents of pupils aged 10–12 (*n* = 45), the most important thing is for the teaching programme in this field to be continuous and systematic, and that knowledge will be constantly consolidated. Parents, regardless of their place of residence, were not in favour of creating a separate teaching subject in this field, especially parents living in the city, because students, in addition to staying at school, often spend a lot of time commuting to school. In their opinion, children are overburdened with learning at school and at home and with extracurricular activities, which is why they do not want them to spend even more time at school. However, this may be based on voluntary participation in extracurricular activities. 

Sample statements by pupils’ parents:

*“If this were combined with one of the existing school subjects, then yes, because children have too many classes already. Another school subject will just burden them. Moreover, if this were an extra, after school class, children would treat it just like an additional lesson. It’d be fun at the beginning, but then it’d be just an unpleasant chore.”*

(parents of pupils aged 10–12, Ostrowiec Świętokrzyski)

*“In my son’s group, there is so much knowledge to absorb, topic after topic, test after test. There’s no room for additional classes.”*

(parents of pupils aged 10–12, Kielce)

*“Children are overstimulated with learning. They have additional extracurricular activities. It would be difficult to find time for nutrition classes.”*

(parents of pupils aged 10–12, Poręba)

The option expected by the vast majority of parents of older children (*n* = 51) is, just as parents of younger children indicated, regular classes dedicated to “healthy” eating as part of existing subjects. The vast majority of parents (*n* = 50) agreed that these classes should take place throughout the entire primary school period and be differentiated according to children’s age. Each year, children should expand their knowledge and learn new, more advanced topics.

All parents participating in the study emphasized that practice, not theory, is crucial. Each time, this topic appeared spontaneously and was discussed with great engagement. According to most parents, regardless of where their children live or what age they are, a combination of practice and theory would bring the best results. During such classes, there should be space to provide theoretical knowledge, as well as for children to ask questions and talk to the teacher. Because of this, pupils would be able to dispel their doubts and explore topics that concern them, rather than just listen to messages imposed on them.

Hands-on activities teach children to be independent in planning and preparing meals and to develop manual skills, especially in case of older pupils. This is especially important because young people are more and more often left alone at home and, many times, they have to eat a meal cooked by their parents or prepare a simple meal themselves. Moreover, parents emphasised that it is important for a child to have an impact on the final content and taste of the dish, as it stimulates their imagination, teaches them creativity, decision-making and independence, and finally encourages them to eat what they have prepared themselves.

Sample statements by pupils’ parents:

*“Only hands-on activities. Theory itself doesn’t guarantee that a child would absorb knowledge, but they would learn it and then they’d forget it.”*

(parents of pupils aged 7–12, Czachówek)

*“After all, you can make great, tasty and ’healthy’ meals from other products. There is a lack of such activities at school.”*

(parents of pupils aged 10–12, Warsaw)

*“In younger year groups, teachers organise such events, prepare simple meals, sandwiches from various products, fruit salads. In older classes, that doesn’t happen anymore. Then there is only theory, learning and passing tests.”*

(parents of pupils aged 7–12, Rosko)

*“They will learn the theory, they’ll pass their exams, and they’ll know that, for example, something contains vitamin D and something else contains vitamin C, but in five minutes they will forget it all. It is not supposed to be just theory, as is the case now, that vitamin C is there, what it is for, how it affects the body. The pupils will learn it, but that’s not the point. It is important that they remember it, more in a practical rather than in a theoretical sense.”*

(parents of pupils aged 10–12, Ostrowiec Świętokrzyski)

Less important for the study participants was who was to be responsible for organising nutrition education at school. The most important thing would be specialist knowledge and interesting ideas on what such classes would look like. Parents of older pupils, just like parents of younger pupils, wish for nutrition education at school to be formalised. This topic would then be treated seriously by schools, parents and pupils, and not as less significant knowledge, extracurricular activities or fun. If it was formalized, the curriculum would also be unified and consistent across all schools.

Sample statements by pupils’ parents:

*“It depends on who will be conducting the classes, because if a teacher comes and says that you need to eat vegetables because they are healthy and then he plays a video about it, because they’re paid for it, I doubt that children will be willing to attend such classes.”*

(parents of a pupil aged 10–12, Rosko)

*“When my son was in primary school in Germany, they conducted a cool experiment. They brought organic and market-sourced vegetables to the classroom. And they observed which ones would go off first. And the children were shocked. And after that Marcel didn’t want to eat supermarket vegetables.”*

(parents of a pupil aged 10–12, Poręba)

Parents of pupils of both age groups agree that nutrition education (meetings, lessons on nutrition) at school should include:

- Lectures by specialists, e.g., dieticians, doctors, nurses;
- Culinary workshops;
- Shows, experiments;
- Trips to production plants, craft workshops, processing plants, e.g., to a local bakery, farmers, orchards, farms, etc.;
- Meetings with role models, e.g., athletes, trainers, dieticians, scientists;
- Visits of guests, e.g., idols from the world of entertainment, music, Internet content creators who can be role models and provide inspiration (e.g., ‘Ekipa’ participants of the Master Chef Junior television show);
- In older classes, a lecture/talk on the prevention of lifestyle diseases, such as diabetes, obesity, hypertension.

### 3.4. Scope and Implementation of Nutrition Education for Parents

Even though parents of younger primary school pupils are aware that the most important stage of nutrition education takes place at home, the vast majority of the participants of this study did not specify whether they would be interested in acquiring knowledge on this subject. In turn, parents of older children responded with moderate openness to the idea of nutrition education dedicated to parents. According to all parents who took part in this research, it all depends on the form, content and schedule of these classes and the topic’s appeal. All parents recognised the importance of such an educational programme. They declared that it is up to them what their children choose to eat, because it is the parents who provide the household with food products and children can make limited choices, choosing from what is available at home. Importantly, the majority of parents participating in the study expect, above all, solutions and assistance that are easy to apply and use in everyday life. They do not want to be required to dramatically change their current way of organising nutrition, including cooking.

Regular lectures devoted to specific topics can encourage at least some parents if the topic is actually important, the method of delivery is interesting, and the form is short and does not require much commitment. Parents from larger cities more often indicated that they would take part in such meetings online. However, the vast majority of respondents stated they would prefer face-to-face meetings combined with hands-on activities.

It is therefore not surprising that the idea of hands-on classes and joint workshops with children on ‘healthy‘ eating sparked enthusiasm among the greatest number of people. This way, they could spend their free time with their children in a pleasant way, learn healthy cooking and discover new solutions and techniques. That might be the result in a kind of ‘Master Chef family‘.

Printed materials such as folders, books and brochures seem to be of little use to parents of both younger and older pupils. They clearly have no time or inclination to read them. Moreover, extensive knowledge in the field of nutrition can be acquired on the Internet; hence, they do not see the need for the creation further publications. Meetings with specialists, such as dietitians and doctors, also did not receive enthusiasm among most of the respondents, least of all in small towns. This form was perceived as boring, not engaging and more suitable for children than adults. It can also be noted that not all parents—especially men from small towns—have trust and respect for dietitians.

The respondents would also like to have access to condensed knowledge that would include:

- Facts to use in a conversation with a child (more practical than theoretical) that would influence their attitude towards eating healthy and nutritious meals; moreover, they should be consistent with the knowledge children receive;
- Results of interesting and important research in the field of nutrition and children’s health in general;
- Sample diets and nutritional patterns appropriate to a child’s age;
- A cookbook or recipes for healthy, nutritious and tasty meals that can be implemented in real life;
- Interesting forms of receiving information such as via a social media profile.

The above-mentioned sources of information are expected to be in the form of a website, mobile phone app or social media profile. Due to the large amount of materials available on the Internet, parents would like to use one, proven, reliable source, e.g., a website authorised by the Ministry of Health. This would build trust, confidence and respect for the information provided. It could also be provided by a charity specialising in this field or a university with nutrition courses, etc.

Overall, the idea of a programme addressed to parents has one main obstacle, namely the need for their involvement. Due to the fast pace of life and many responsibilities, parents are afraid that they would be required to devote more time and energy to additional topics that are not always pleasant or interesting for them. 

Sample statements by pupils’ parents:

*”That’s a problem. Some parents think they don’t need it, others think they know everything, and others think it’s not an important issue. The question is who it’d be for and how to encourage parents? Maybe, for example, we could cook something together with a child? In order to eat something tasty?“*

(parents of pupils aged 7–12, Warsaw)

### 3.5. Nutrition Education for Parents versus Parents’ Attitudes towards Nutrition

**Figure 1:**
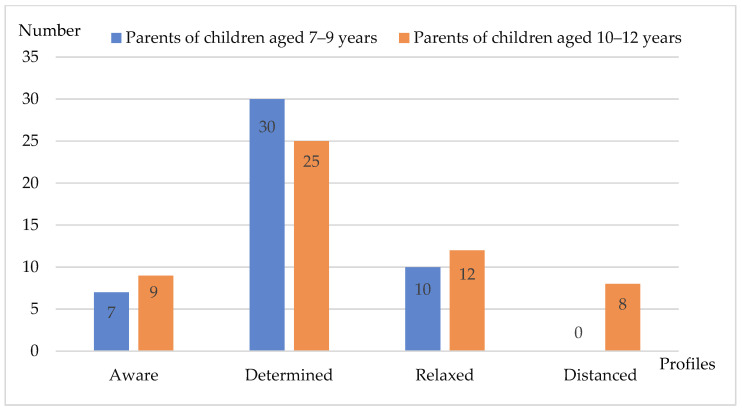
The profiles of pupils’ parents [*n* = 101].

Due to the fact that pupils’ parents differ in terms of their knowledge and attitude towards nutrition for themselves and the entire family, the following parent profiles were identified: ‘aware’, ‘determined’, ‘relaxed’, ‘distanced’ (Figure 1), depending on their attitudes, and interest in forms of nutrition education assigned to them (Table 2).

Parents classified as the ‘aware’ profile (*n* = 16) show characteristic features in their approach to food and nutrition. They pay special attention to the eating habits of the entire family, especially children. They are interested in topics related to nutrition and its impact on health and human body. Moreover, they enjoy it. These are most often women living in cities and larger rural areas near large urban centers. The largest group are parents (*n* = 55) classified as the ‘determined’ profile. They are both women and men from rural and urban areas, with the largest group being residents of larger cities. Parents’ approach to nutrition varies depending on the age of the children, with parents of older pupils having a more relaxed approach to the topic of nutrition. In turn, parents of younger children feel more tension and stress related to nutrition. Both groups of parents are equally committed to ensuring that their children adopt healthy eating habits, but it is a challenge for them. Parents belonging to the ‘relaxed’ profile (*n* = 22) are often men living in smaller towns or rural areas. They realize how important proper nutrition is, but they believe that it is a luxury reserved for those who have a lot of free time. These parents understand that they have limited nutritional knowledge. In their opinion, schools should be responsible for children’s nutritional education. In turn, the profile of a ‘distanced’ parent (*n* = 8) is specific only to parents of older pupils (10–12 years old). These are mainly women from large cities who are specialists in their fields and their intensive career is their priority. They believe that children at school should primarily focus on acquiring scientific knowledge to ensure a good education. In their opinion, nutritional education is not knowledge, but rather a matter of habits and customs that can be easily shaped. More details about pupils’ parents’ profiles were described previously [29].

Taking into account the profiles of the parents of pupils (both younger and older) identified as part of FGI in terms of the level of involvement and approach to the topic of nutrition, we notice that the parents with the ‘determined‘ profile are the most willing to be educated. This is due to the fact that they have the biggest gaps in their own knowledge but at the same time the greatest willingness to pass on their knowledge to their children, and they are also ready to change both their own and their children‘s eating habits.

The least likely to participate are ‘relaxed‘ parents, who neither find time nor need to take part in this type of activity offered by school, because they have a rather relaxed attitude towards proper nutrition. This approach is also shared by a group of parents of children aged 10–12 defined as ’distanced’. This term can be used to describe parents who show no interest in food or nutrition issues. Parents from both of these profiles are not ready to listen to authority figures in the nutrition field and feel no need to do so, because they believe that they know best their children and their needs in terms of food and nutrition.

In turn, the parents participating in this research and representing the ’aware ’ profile already have such extensive knowledge in this field that the proposed topic and form would have to be truly engaging and innovative. However, they would generally be interested in taking part in a programme aimed at parents. When it comes to the type of materials or form of education, the most popular would be theoretical and hands-on classes, while other forms of learning met with mixed attitudes from the respondents, which depended on their level of knowledge and on how much time they would need to devote to learning. 

### 3.6. Educational Materials and Communication Needs of Primary School Pupils—Parents’ Expectations and Ideas

According to the majority of parents (*n* = 69) participating in this study, the role of social media, influencers, YouTubers and bloggers in acquiring knowledge about nutrition can be enormous and is crucial in the case of older pupils. In the parents’ opinion, content on healthy eating could be provided via virtually every information and entertainment medium used by children and teenagers, i.e., games, clips, cartoons, educational shows, content provided by Internet creators, etc. The conditions that would have to be met are interesting and engaging content, a captivating script, the right pace and a regular schedule. The form would need to be fairly short with attractive graphics (colourful, modern) so that pupils would not get bored and they would not switch off and choose a different video. The message might be well reinforced by the presence of role models from the world of sports, music or games and by Internet content creators.

The opinions of the parents of younger and older pupils were divided regarding the traditional form of educational materials. Yes, parents would like their children to read paper versions, e.g., books, comics, brochures, etc., but at the same time, they openly admit that they seem to be the least engaging for children. They quickly get bored and, as a result, they forget about them, put them on the shelf, and, after some time, throw them in the bin. The advantage of online solutions is also greater interactivity and the ability to constantly provide new content, organise competitions, insert photos, ask questions, etc. This is an important aspect emphasised by parents because, in their opinion, education should be two-dimensional, i.e., both provider and user should have an active influence on the content conveyed.

The idea of a mobile phone app dedicated to nutrition education emerged among the parents of pupils aged 10–12. Nowadays, parents allow their children to use similar solutions, e.g., to learn foreign languages. Another interesting solution seems to be a game for children, in which they decide what the character eats and what products they choose, and then they observe what happens to this character (the impact of nutrition on behaviour, energy level, physical fitness, concentration, etc.). Some individual respondents expressed their concern and reluctance towards online solutions, even those promoting healthy eating. They were worried that their children would spend even more time in front of a computer screen or using a mobile phone. An interesting idea that parents found engaging is an educational game in an online and offline version, i.e., a board game. It would be a way to spend time as a family, and broaden their knowledge on nutrition.

## 4. Discussion

The main authority and source of knowledge in the field of nutrition for primary school pupils, especially for those in the first elementary classes, are their parents. Their role in shaping children’s eating attitudes is not only to provide information on food and nutrition, but above all to set the right example of specific eating behaviours consistent with the knowledge transferred in order to prevent cognitive dissonance among children.

Our research demonstrated the need for nutrition education which aims to promote regular consumption of meals, increase the consumption of vegetables and fruit, and limit the consumption of sweets, sugar and sweetened drinks. Moreover, among older children, in the opinion of their parents, the scope of education should concern energy drinks, fast food and salty snacks, as well as the ability to read labels, the choice of unprocessed food, energy values, the use of various diets, diet-related diseases, zero waste, and food production methods.

Parents emphasised the need for formal school nutrition education, especially in the younger group (preschool education). This is consistent with the reports of other authors. It has been demonstrated that school nutrition programmes contribute to improving nutritional knowledge as well as increasing the consumption of certain groups of products, especially vegetables and fruits. However, this requires long-term educational solutions as well as adequate transfer of knowledge and practical skills [35,36,37]. Such solutions are more effective than family-based preventive nutrition and physical activity interventions, which have challenges such as difficulties in recruiting and retaining families throughout the programme. Research results prove the low effectiveness of these programmes [38,39,40]. An additional problem observed among parents of overweight or obese children is the fear of stigmatisation of children who cannot lose weight [41]. However, the authors emphasise that it is necessary to take into account the overloaded curricula and the number of teachers in primary schools, as well as the limited financial resources for undertaking such programmes [36].

Moreover, many barriers affecting the effectiveness of education have been identified, e.g., regarding the consumption of vegetables and fruit by children. The most common barriers include neophobia, taste preferences, lack of self-efficacy, time constraints, insufficient cooking skills and low social support, motivation and willpower [42,43]. Therefore, this should be taken into account when introducing new programmes and assessing their effectiveness. It has also been shown that there is often a lack of awareness that, for example, the consumption of vegetables is too low in relation to the recommendations [42]. Therefore, the acquisition knowledge by pupils and their parents is the first step to changing their eating behaviours [44], which was also noticed in our research.

In our research, parents indicated the need for more workshops and hands-on activities, with a theoretical foundation related to the scope of practical activities. And although they were quite sceptical about their own nutrition education, they would be happy to take part in culinary workshops with their children, which would allow them to gain new skills and to learn to prepare tasty, healthy and visually attractive meals.

A review of studies on educational programmes addressed to school-age children confirms that learning through cooking (children preparing dishes themselves) may have a beneficial effect on improving psychosocial factors, including eating preferences, habits and behaviours [45,46,47]. As a result of such educational activities, it was noticed, among other things, that there was an increase in fiber consumption, increase in fruit and vegetable consumption, in willingness to cook, in willingness to try unknown foods, and in acceptance of vegetables [48]. Other authors also point out the beneficial impact of nutritional education on eating behaviour, including the improvement of anthropometric parameters [45,49,50]. An important aspect is also the inclusion of activities promoting increased physical activity and reducing so-called screen time. Due to the growing percentage of obese children and adolescents, it seems important to create favourable conditions for increasing physical activity for all children, regardless of place of residence or socioeconomic status, and to increase access to free public recreation spaces, especially outdoors [51]. These aspects were also emphasized by the parents of the children in our study.

To increase the impact of the programme, especially on dietary behaviors (i.e., fruit and vegetable intake, breakfast consumption) and eating skills, it may also be important to consider psychological aspects. Hence, it is worth paying attention to the CAN method, which, developed by a consumption psychologist, is a model that facilitates the introduction of dietary changes [52] based on three principles: convenient access—healthy food should be the easiest choice; attractive choice—improving the attractiveness of a healthy product through its name or appearance; and normal behaviour—if healthy snacks are more available than unhealthy snacks, it is clear what the norm is. Taking into account that about 200 decisions on food are made during the day [53], it is therefore difficult to choose the right products, and it is even more important that when making a choice, the instinctive choice is healthy food, not sweets or salty snacks [54]. It is believed that prohibiting or restricting access to unhealthy products is less effective than choosing attractive, healthy foods [55,56]. The attractiveness of products with health-promoting properties can be improved through their name, appearance and method of serving [57,58,59].

Many authors point out that parents’ knowledge and behaviour have a significant impact on children’s eating behaviour. A connection has been found that the food consumed by parents is a model for children’s eating habits [60]. Generally, a better quality of parental diet was associated with lower consumption of cakes, chocolate, biscuits and also savoury foods by children [61]. The use of food as a reward, especially unhealthy, high-energy and highly flavoured (because of the content of e.g., sugar) food, is also considered as undesirable parental behaviour [62] in the same way as very strict restrictions, which makes these foods desirable [62,63]. Therefore, it is not recommended to apply pressure, as it may create a negative eating environment in the family and make children picky eaters [64]. In these children, this may lead to the development of nutritional abnormalities, including neophobia and eating disorders [65].

Similarly to our own study and previously studies, it was shown that the preparing and eating of meals by a family together was considered a key strategy for promoting proper nutrition, which may consequently translate into the development of healthy eating habits and the prevention of obesity among children [66]. “Guidance and encouragement” eating practices used by parents can help their children develop healthier eating habits, including choosing appropriate foods and meals [63]. It has also been stated that attractive, educational nutrition programmes intended for a wider group of children can compensate for differences resulting from origin or other social inequalities, and the active involvement of parents is crucial for the success of an intervention and in reducing the risk of excessive body weight in children [67].

Our own research and that of other researchers [47,67] shows that education of parents seems to be necessary, although they themselves, as shown in the studies below, are quite sceptical about this, pointing to time constraints and their children’s extracurricular activities. Nevertheless, they mentioned where educational activities could take place, e.g., on social media, on websites dedicated to parents and in the form of mobile phone apps. However, they would most like to participate in hands-on culinary workshops with their children. Parents also see schools as the best source of nutrition education; it is recommended that culinary programmes be integrated into the schools’ curriculum.

This is also emphasised by other authors who suggest that the teaching of nutrition education in primary schools by qualified teachers can make a significant contribution to children’s knowledge and eating habits [68]. Some authors also point to the effectiveness of even short-term education of parents in improving their own eating behaviours, which results in parents starting to choose more healthy food options for their children [69,70].

The use of qualitative research using the FGI method allowed for spontaneous statements about food and nutrition, as well for obtaining ideas regarding nutrition education from pupils’ parents. In the future, this will allow us to create appropriate strategies to promote ’healthy’ nutrition for children and adolescents in order to prevent the consolidation of inappropriate eating habits for life. Childhood and adolescence are important periods for the development of eating habits due to changes in eating skills during these periods, including modifications in eating behaviours, as well as physiological, cognitive and social changes. However, it should be remembered that strategies for promoting proper nutrition should be adapted to the child’s stage of development and the cultural context, as well as to generational change [1].

## 5. Conclusions

Our research demonstrated that the main authority and source of knowledge in the field of nutrition for primary school pupils, especially for those in the first elementary classes, are their parents and, for the group of older children, also peers. Therefore, it seems reasonable to develop a multi-aspect nutritional education programme covering children, their parents and teachers. The need for coherent presentation of educational content addressed to teachers and parents is very important, so that they can uniformly shape attitudes towards food and nutrition of children and adolescents.

Moreover, cooperation between school and family may result in greater effectiveness in promoting proper nutrition. Planning an appropriate strategy, its individual elements, techniques and methods, as well as various activities in the school environment, involving both children and their parents, can consolidate correct eating behaviors learned at home or help correct incorrect eating habits. Furthermore, schools can have more of an impact on correct eating behaviours by creating a positive image of ’healthy’ food.

## Figures and Tables

**Table 1:** Pupils’ needs in the field of nutrition education in the opinion of parents.

Topic	Importance for Pupils *		Important Aspects from the Point of View of	
	Aged 7–9	Aged 10–12	Parents of Pupils Aged 7–9	Parents of Pupils Aged 10–12
Sweets, sugar, sweetened drinks	5	5	dangers and effects of consuming large amounts of simple sugars; the amount of sugar in children’s favourite products (sweet snacks, etc.); allowed amount of sugar per day/kg of child’s body weight; what to substitute for sugar; what to do when a child wants something sweet to eat; how to eliminate excess sugar in the diet (methods, techniques, tips, etc.); how to prepare a simple, homemade dessert with reduced or zero sugar content	
Daily intake of vegetables and fruits	4	5	why fruits and vegetables are so important in a child’s diet; what a portion of fruit or vegetables is and how many portions should be eaten per day; which vegetables are best for health due to their nutritional composition; which fruits are best for health based on the glycemic index; what time of day fruit should be eaten; optimal size of the proportion of vegetables and fruit on the plate	
Meals during the day	4	5	how many and what meals pupils should eat during the day; what the role of each meal and its importance for the body’s health and development is; the consequences of skipping meals; what the optimal meal schedule for a child is, what happens if a child eats dinner too late or too early before going to bed	
Portion size and product proportions	5	5	optimal portion size (what a portion means, the knowledge to select the quantity and proportion of products on the plate); what proportion of a given group of ingredients should be in a meal (protein, carbohydrates, fats, etc.); how to prepare meals	
Beverage intake	5	2	the importance of hydrating the body and its impact on body functions; correct drinking schedules (how many liters a day, in what portions); what is the best way to quench your thirst	
Nutritional ingredients	4	4	nutrients (fat, protein, fiber, vitamins, etc.), their sources and role in the body; effects of excess and deficiency of nutrients	
Fats	3	3	types, role, fat substitutes (e.g., yoghurt instead of mayonnaise)	
Wholegrain products	3	3	what and how often to eat; why they are important in the diet; what nutrients they provide	
Milk and dairy products	3	2	what and how often to eat; their role in the diet; nutrients provided	
Fast food and salty snacks	5	5	dangers to the body, what ingredients they contain, alternative substitutes for snacks and fast food	
Physical activity	3	4	what form of exercise is recommended for pupils; how the body behaves in the absence of movement and physical exercise, what the impact of movement is, how it supports the body’s proper development	
			not applicable	why e-sports (electronic sports) are a threat
Labels	---	3	not applicable	why and how to read food labels
Processed food	---	4	not applicable	the advantage of fresh products over processed ones; functional food additives (preservatives, dyes, etc.), their role, harmful effects
Diets	---	4	not applicable	truths and myths about weight loss diets (crucial for girls); principles and implementation of vegetarian and vegan diets
Calorie content of products	---	3	not applicable	what the optimal amount of calories per day is (energy requirement for children)
Energy drinks	---	5	not applicable	why you should not drink energy drinks; what ingredients they contain and how they affect the body
Lifestyle diseases	---	5	not applicable	what the symptoms are and how to prevent diabetes, obesity, hypertension, etc.; basic information on diet-related diseases

* scale: 5—very important, 4—important, 3—moderately important, 2—not very important, 1—not important.

**Table 2:** The respondents’ interest in forms of education for pupils’ parents.

Criteria	Pupils’ Age	Parents’ Profile versus Their Interest in Nutrition Education			
		‘Aware’ *n* = 16	‘Determined’ *n* = 55	‘Relaxed’ *n* = 22	‘Distanced’ *n* = 8
		Level of Interest in Nutrition Education for Parents			
general interest in nutrition education for parents	7–9 years old	medium	high	low	does not apply
	10–12 years old	medium	high	low	low
printed materials (e.g., folders, books, booklets)	7–9 years old	low	medium	low	does not apply
	10–12 years old	low	medium	low	low
regular lectures on a specific topic/problem	7–9 years old	medium	high	low	does not apply
	10–12 years old	medium	high	low	low
meeting with specialists, i.e., dietitians, doctors	7–9 years old	low	medium	low	does not apply
	10–12 years old	low	medium	low	low
combined hands-on and theoretical classes	7–9 years old	high	high	medium	does not apply
	10–12 years old	high	high	medium	low
social media content	7–9 years old	high	high	low	does not apply
	10–12 years old	high	high	low	medium
website	7–9 years old	high	high	medium	does not apply
	10–12 years old	high	high	medium	low

## Data Availability

Data are contained within the article and Supplementary Materials.

## Supplementary Materials

The following supporting information can be downloaded at: https://www.mdpi.com/article/10.3390/nu16010129/s1, Supplementary File S1: Focus Group Interview scenario.
