# Supplementary material for: How Can We Increase the Nutrition-Related Knowledge in Children Aged 7–12 Years: Results of Focus Groups Interviews with Parents—Junior-Edu-Żywienie (JEŻ) Project
Source: Nutrients. 2023 Dec 30;16(1):129. doi: 10.3390/nu16010129 (PMC10780913; doi:10.3390/nu16010129)
Supplement: Supplementary file 1 [file nutrients-16-00129-s001.zip › nutrients-2733853-supplementary.pdf]

# "FOCUS GROUP INTERVIEW SCRIPT AMONG PARENTS of PRIMARY SCHOOL CHILDREN AGED 7-9 YEARS OLD (and AGED 10-12 YEARS OLD)"

**Participants of the interview:** 6-8 parents of primary school children aged 7-10 years old (aged 10-12 years)

**FGI duration:** 90 minut

| |
|---|
| **1. Introduction to the group discussion (5 minutes)** |
| • Moderator's introduction<br>• Presentation of the research purpose and the purpose of conducting the group interview<br>• Explanation of the rules for conducting the group interview<br>• Explanation that the focus group interview will be recorded, and the recorded content is confidential and will be used for research purposes<br>• Asking participants if they have any questions - moderator's responses and clarification of any doubts<br>• Informing about the duration of the meeting: up to 1.5 hours |
| **2. Evaluating parents of children's approach to nutrition (5 – 10 minutes)** |
| How important is the topic of proper nutrition, diet, valuable products, etc. in your homes/families? We wait for a spontaneous answer and then ask:<br>• do you like these topics? Are you happy to discuss them?<br>• How important do you think they are?<br>• How often do you talk to your children about these topics?<br>• Are children interested in these topics?<br>• From your point of view, are these topics easy?<br>*The moderator can ask the following questions in each topic:*<br>- Why yes / no?<br>- What is the reason for this? |
| **3. The current state of nutritional education for children in the opinion of parents (15-20 minutes)** |
| How do you assess the nutritional knowledge level of your children (grades 1-6) in terms of:<br>• rules of proper nutrition?<br>• food products?<br>• methods of food production?<br>*The moderator can ask the following questions in each topic:*<br>• why do you think so?<br>• what does your answer mean?<br>• What information do children particularly lack in this area?<br>Are these topics important from your point of view?<br>- why do you think so?<br>- what does your answer result from?<br>Do you think that current curricula include content related to:<br>- rules of proper nutrition?<br>- food products?<br>- methods of food production? |

*The task entitled 'Conducting scientific research in the field of nutrition of children and adolescents, developing and implementing a nutritional education program for pupils of grades 1-6 of primary schools. Acronym: Junior-Edu-Żywienie (JEŻ)'. The study was financially supported by the Polish Ministry of Education and Science (MEiN/2022/DPI/96 of March 7, 2022).*

If so, in which classes are these topics most often discussed?

Do you think that food and nutrition topics should be included in teaching curricula?

- Why yes / no? why do you think so?
- What benefits do you see from discussing such topics at school?
- What weak points do you notice when discussing such topics at school?

- What specific areas related to nutrition do you think should be included in school curricula?
- What knowledge does your children lack? What problems do you struggle with your child's diet and nutrition?

*The moderator collects spontaneous answers, and if they do not appear, we also ask about:*

- sweets, sugar, sweetened drinks
- energy drinks
- dairy products
- whole grain cereal products
- daily intake of vegetables nad fruits
- optimal numer of meals per day
- optimal time to eat meals during the day
- portion size and proportion of ingredients/ product groups
- liquid intake during the day
- fast food
- salted snacks
- physical activity
- nutrients, sources and roles of: fats, calcium, vitamins, fiber, protein, etc.
- vegan diet

*The moderator asks briefly:*

- How important is this topic for you? What it comes from?
- Why is this topic problematic in your home/ in your children's nutrition?
- Which aspects are particularly important and therefore there should be more education about them?

**4. Sources of information among students from the perspective of parents (10-15 minutes)**

- Where do children get knowledge about topics that interest them? (we ask generally to identify the sources most relevant to them)
  - channels of famous youtubers: what specific names?
  - Influencers: jakie konkretne osoby/ nazwiska/ pseudonimy?
  - Facebook, Instagram: what specific profiles?
  - Tik-tok: what specific profiles?
  - blogs: what specific names?
  - peers
  - open and closed groups on social media: what topics and main thread?
  - youth press: what titles?
  - books and guides: what titles?
  - teachers
  - parents
  - TV programs: what specific names?

***The task entitled 'Conducting scientific research in the field of nutrition of children and adolescents, developing and implementing a nutritional education program for pupils of grades 1-6 of primary schools. Acronym: Junior-Edu-Żywienie (JEŻ)'. The study was financially supported by the Polish Ministry of Education and Science (MEiN/2022/DPI/96 of March 7, 2022).***

- What is the role of social media, influencers, youtubers and bloggers in students' acquisition of nutritional knowledge?
  - Do you think it can also be an important source of knowledge also in the field of nutrition? Why is this possible or impossible?
  - What conditions would have to be met for these to be important sources of knowledge? Interesting content, appropriate intensity, attractive graphics?

**5. The scope and implementation of nutritional education for children in the opinion of parents (10-15 minutes)**

- What do you think nutritional education for children at school should look like?
  - a separate teaching subject
  - talks during educational classes with class teacher
  - extra teaching hours
  - meeting with specialists: dietetitians, doctors, athletes, etc.
- How long do you think nutritional education should be continued at school? (a few hours a year, a whole semester, several years)?
- Should nutritional education at school be differentiated depending on the age of children? What age groups do you propose to divide students into? What it comes from?
- Should classes be only theorethical or should they also include practical classes? Which one for example? What should children learn during such classes?
- Who should be responsible for nutritional education at school? School director, class teacher, nurse, external institution, who else?

**6. Scope and implemetation of nutritional education for parents (5-10 minutes)**

- Do you think that nutritional education for students' parents should be provided in primary school? If so, in what way (methods)? What form would be attractive to you?
  - meeting with specialists: dietitians, doctors, athletes, etc.
  - cyclical lectures devoted to specific topics/problems
  - theoretical and practical classes for parents and children (together)
  - online form/offline
  - printed materials: folders, books, brochures, research results, sample diets and nutritional patterns
  - practical activities

**7. Educational materials and parents' communication needs (10 minutes)**

- What is needed to implement nutritional education? For example, availability of materials for parents and children, that increase the level of knowledge? More pressure from the school/ Ministry of National Education/ Ministry of Health?
- What media should be used in children's nutritional education? (TV, radio, social media)
- What publications addressed to children should be prepared as part of nutritional education (books, guides, comics, etc.)?
- What form of educational materials is the most beneficial?
  - printed
  - multimedia
  - video films etc.
- What additional teaching aids should be prepared for nutritional education to be effective? Do

*The task entitled 'Conducting scientific research in the field of nutrition of children and adolescents, developing and implementing a nutritional education program for pupils of grades 1-6 of primary schools. Acronym: Junior-Edu-Żywienie (JEŻ)'. The study was financially supported by the Polish Ministry of Education and Science (MEiN/2022/DPI/96 of March 7, 2022).*

you have any specific ideas?

- Should social media be used in nutritional education and how? Do you have any specific ideas?

**8. The pandemic and dietary habits (5 minutes)**

- Do you still remember the times of the pandemic? Your children couldn't go to school, and education was distance, so you probably spent most of your time at home with children.
- What particularly changed in your life then?
- How did your daily routine change during that time? What changes occurred?"
- How did you feel?
- How did you spend your time?
- Did you engage in any additional activities?
- Do you feel that the whole family had more or less physical activity?
- Do you feel that you ate different foods or meals than usual during that time? What foods do you associate with that period?
- What types of foods did you eat more? Do you still eat those products? Why yes/no?
- What types of foods did you eat seldom?
- Do you feel that you are eating in the same way as during the pandemic?

**9. Summary of the group discussion (5 minutes)**

- A brief summary of the discussion.
- Is there anything else anyone would like to add?
- Thank you for participating in interview.

*The task entitled 'Conducting scientific research in the field of nutrition of children and adolescents, developing and implementing a nutritional education program for pupils of grades 1-6 of primary schools. Acronym: Junior-Edu-Żywienie (JEŻ)'. The study was financially supported by the Polish Ministry of Education and Science (MEiN/2022/DPI/96 of March 7, 2022).*
